# Comparison of Methods for miRNA Extraction from Plasma and Quantitative Recovery of RNA from Cerebrospinal Fluid

**DOI:** 10.3389/fgene.2013.00083

**Published:** 2013-05-16

**Authors:** Melissa A. McAlexander, Maggie J. Phillips, Kenneth W. Witwer

**Affiliations:** ^1^Retrovirus Laboratory, Department of Molecular and Comparative Pathobiology, The Johns Hopkins University School of MedicineBaltimore, MD, USA

**Keywords:** miRNA, biomarker, biofluid, RNA isolation, method, RT-qPCR, plasma, cerebrospinal fluid

## Abstract

Interest in extracellular RNA (exRNA) has intensified as evidence accumulates that these molecules may be useful as indicators of a wide variety of biological conditions. To establish specific exRNA molecules as clinically relevant biomarkers, reproducible recovery from biological samples and reliable measurements of the isolated RNA are paramount. Toward these ends, careful and rigorous comparisons of technical procedures are needed at all steps from sample handling to RNA isolation to RNA measurement protocols. In the investigations described in this methods paper, RT-qPCR was used to examine the apparent recovery of specific endogenous miRNAs and a spiked-in synthetic RNA from blood plasma samples. RNA was isolated using several widely used RNA isolation kits, with or without the addition of glycogen as a carrier. Kits examined included total RNA isolation systems that have been commercially available for several years and commonly adapted for extraction of biofluid RNA, as well as more recently introduced biofluids-specific RNA methods. Our conclusions include the following: some RNA isolation methods appear to be superior to others for the recovery of RNA from biological fluids; addition of a carrier molecule seems to be beneficial for some but not all isolation methods; and quantitative recovery of RNA is observed from increasing volumes of cerebrospinal fluid.

## Introduction

A leading goal of modern biomarker studies is to ascertain disease conditions and other biological states by examining molecules in easily obtained biological fluids. For example, the ability to use blood-based markers for diagnosis, location, and staging of cancer, or to predict outcome of several therapeutic options, would have several benefits, from early detection to obviating the need for invasive and expensive biopsies. While some biomarkers may simply be “bystanders” in the disease process, others might be actively involved and thus present targets for novel therapeutics (Kota et al., 2009; Cho, 2012; Lindow and Kauppinen, 2012). Of course, any successful marker or set of markers would need to be sufficiently stable during and following the isolation process to allow reliable detection and measurement. Among the many types of biomolecules currently under investigation as potential biomarkers are extracellular RNA (exRNA), including microRNA (miRNA) (Pritchard et al., 2012a). These short oligonucleotides are thought to be stable in extracellular fluids, protected from degradation by ubiquitous RNases (Mitchell et al., 2008) within or even on the surface of small particles.

Many questions about extracellular miRNAs are under active investigation. The identities of the various particles that carry and protect miRNAs, the frequency of miRNA association with different particle types, and the relative contributions of these particles to exRNA profiles in health and disease have yet to be firmly established (Witwer et al., 2013). The extent to which miRNAs are specifically or non-specifically associated with particle types is also largely unknown. Finally, the extent and mechanisms of function of extracellular miRNAs – e.g., in intercellular signaling – remain incompletely understood, although numerous studies have established that such functions exist (Valadi et al., 2007; Pegtel et al., 2010; Ismail et al., 2012). In the face of these many outstanding questions, the current consensus is that extracellular vesicles (Lotvall and Valadi, 2007), protein complexes (Arroyo et al., 2011; Turchinovich et al., 2011), and, possibly, additional particles (Turchinovich et al., 2012), contribute to the extracellular population of miRNAs. Furthermore, specific extracellular miRNAs or constellations of miRNAs appear to be co-regulated during disease: to give just a few of many examples, in cancers (Cho, 2011), acute retroviral infection (Witwer et al., 2011), and autoimmune disease (Murata et al., 2010).

The promise of extracellular miRNA biomarkers is tremendous, but several obstacles must be surmounted during ongoing, successful development of miRNA signatures of disease and other conditions. First, many high-profile preclinical studies in general cannot be replicated or reproduced (Ioannidis, 2005; Ioannidis et al., 2009); in the same way, many miRNA profiling studies are underpowered or analysis cannot be replicated because data are missing or procedures inadequately explained (Baggerly, 2013; Witwer, 2013). In some cases, then, better study design and reporting would be helpful. Second, lack of standardization is a challenge to much-needed comparisons of miRNA studies. Biological fluids contain small amounts of RNA relative to cells and tissues; if sample processing does not remove small cells and cell fragments, miRNAs from these particles may predominate in any “extracellular” miRNA profile (McDonald et al., 2011), just as RNA from hemolysed samples may affect profiling (Kirschner et al., 2011; Pritchard et al., 2012b). A study of miRNAs in platelet-rich plasma – say, plasma that has been spun for 10 min at 800 × *g* – might simply reflect disease state-associated platelet abundance. Third, because of the aforementioned relative paucity of RNA in cell-free fluids as well as the presence of PCR inhibitors in biological fluids, efficient extraction methods are needed to ensure maximum recovery of RNA from low volumes of input fluid.

While several studies have described methods for RNA extraction from cell-free fluids (Tzimagiorgis et al., 2011; Debey-Pascher et al., 2012) or total blood (including cells) (Gaarz et al., 2010), relatively few studies have specifically compared the use of commercially available kits for RNA extraction from biofluids (Burgos et al., 2013) or constituents of biofluids (Eldh et al., 2012). We are unaware of any such studies employing recently released biofluids-specific RNA extraction methods from Qiagen and Exiqon. Here, we report comparisons of five commercially available kits/methods for RNA extraction from plasma; the effects of including or omitting glycogen as a carrier/co-precipitant; and the linear recovery of RNA from increasing volumes of plasma and cerebrospinal fluid (CSF) using a biofluids-specific method.

## Materials and Methods

### Ethics statement

De-identified plasma was from a healthy donor who provided informed consent in accordance with approval by the Johns Hopkins Institutional Review Board. CSF was from a healthy donor pigtailed macaque (*Macaca nemestrina*). Animal studies were approved by the Johns Hopkins University Institutional Animal Care and Use Committee and conducted in accordance with the Weatherall Report, the Guide for the Care and Use of Laboratory Animals, and the USDA Animal Welfare Act. Macaques are pair- or group-housed when possible and receive environmental enrichment including manipulanda, foraging, and opportunity to exhibit species-specific behavior. Animals are continually monitored for signs of distress. Ketamine is administered for blood or CSF draws.

### Reagents

Paired stem-loop reverse transcription primers and hydrolysis probe/primer combinations for RT-qPCR were purchased from Life Technologies for the following miRNAs: endogenous mammalian miRs-16, -21, -34a, -126, and -150, and exogenous cel-miR-39. These miRNAs, along with most relatively abundant miRNAs, are conserved amongst primates (Brameier, 2010; Shao et al., 2010). RNA isolation kits were from Life Technologies (mirVana, AM1561), Exiqon (miRCURY RNA Isolation Kit – Cell and Plant, #300110; and miRCURY RNA Isolation Kit – Biofluids, #300112), and Qiagen (miRNeasy Serum/Plasma Kit, # 217184). TRIzol LS reagent was purchased from Life Technologies (#10296-010). TaqMan microRNA Reverse Transcription Kit (#4366596) and qPCR master mix (#4440047) were purchased from Life Technologies. iScript select cDNA synthesis kit (1708897) and SsoFast qPCR mix (1725232) were from BioRad. Additional reagents included glycogen (Ambion/Life Technologies, AM9510), synthetic cel-miR-39 (Integrated DNA Technologies – custom product synthesized to sequence from miRbase.org) (Mitchell et al., 2008), and highly pure diethyl pyrocarbonate (DEPC)-free and nuclease-free water (Qiagen, 129115).

### RNA handling and storage

We followed RNase-free protocols throughout all procedures up to qPCR setup (after RNA has been converted to cDNA). A laminar air flow cabinet dedicated to RNA work was used for RNA isolation and reverse transcription preparations. Surfaces (cabinet, racks, pipettes, centrifuge) were cleaned thoroughly with RNase-away prior to and after use. Pipettes and other portable objects to be used in the hood were exposed to UV light in a UV crosslinker for 2 min on each side. This was done to avoid introduction of contaminating nucleic acids and, potentially, proteins. We implemented double-gloving, regular glove changes, and wearing of Tyvek sleeves or clean lab coats. Certified RNase-free barrier pipette tips were used. Where possible, all reagents were certified RNase-free. Isolated RNA from all isolation methods was stored at −80°C.

All RT-qPCR reactions were performed with once-thawed RNA as substrate. RNA samples were thawed on ice, mixed gently, and centrifuged at 2500 × *g* for 5 min in a table-top centrifuge before use. This was done because some column-based clean-up methods introduced a fine white powder into the eluted sample, presumably from the filter materials. Although this material is likely inert, we pelleted it to avoid interference with downstream reactions. It may be preferable to pellet this material and transfer the clarified supernatant to a new RNase-free tube prior to initial freezing.

### Sample handling

RNA isolations were performed from platelet-poor primate plasma that had been frozen (−80°C) and thawed once. Platelet-poor plasma was defined as plasma separated from blood cells less than 30 min following blood draw with a 1000 × *g* spin for 15 min at room temperature followed by a 2500 × *g* spin for 15 min at room temperature to remove most remaining platelets. Plasma was obtained from blood collected into ACD anticoagulant. All methods comparison experiments were conducted with aliquots of plasma from a single blood draw from one donor.

### RNA isolations

For all isolations, 5 or 10 pg synthetic cel-miR-39 per isolation was spiked into the respective lysis/denaturant buffer before combining with plasma. We note that synthetic RNA cannot be added directly to plasma or other biological substances because endogenous RNases degrade it immediately (Mitchell et al., 2008). Also, fresh cel-miR-39 dilutions were prepared for each set of isolation experiments since storing highly diluted RNA could result in substantial loss of material to container surfaces. Three micrograms highly pure glycogen (as indicated) per isolation was either omitted (negative) or added (positive) as a carrier/co-precipitant. We emphasize the importance of mixing samples well (including, where appropriate, vigorous vortexing for at least 1 min prior to centrifugation for phase separation). Screw-cap tubes are recommended to minimize losses during organic extraction methods.

#### Comparison of three RNA isolation methods, with and without glycogen

To compare mirVana, miRCURY Cell and Plant, and TRIzol LS isolation with mirVana column clean-up, triplicate isolations for each method were performed from 100 μl plasma each, both with and without glycogen (see above) for a total of 18 isolations. Manufacturer’s protocols were followed for the Exiqon and Ambion/Life Technologies kits. For TRIzol LS/column clean-up, 100 μl plasma was diluted with 150 μl nuclease-free water, followed by addition of 750 μl TRIzol LS reagent. Samples were mixed and incubated at room temperature for 10 min. Two-hundred microliters chloroform was added with a glass pipette, followed by vortexing for 1 min and incubation at room temperature for 5 min. Phases were separated by centrifugation at 14,000 × *g* for 15 min in an Eppendorf 4514C centrifuge. Aqueous layer was removed, and 1.5 volumes EtOH were added. This mixture was applied to mirVana kit filter columns followed by wash and elution steps as per manufacturer’s protocol. For this isolation experiment, the miRNAs measured were endogenous miR-16 and miR-21 and spiked-in cel-miR-39. miRNAs were measured by RT-qPCR in triplicate for each isolation. “Fold abundance” was calculated in comparison with the average of results for the three no-glycogen mirVana isolations.

#### Comparison of Exiqon miRCURY cell and plant kit and Exiqon miRCURY biofluids kit

Duplicate isolations from 100 μl plasma were done for each method. Glycogen was added for isolations using both methods; a no-glycogen condition was included only for the Biofluids Kit, for a total of six isolations. The manufacturer’s protocols were followed. miRNAs measured included endogenous miRs-16, -34a, and -126, and spiked-in cel-miR-39. miRNAs were measured by RT-qPCR in triplicate for each isolation. “Fold abundance” was calculated in comparison with the average of results for the two glycogen-added miRCURY Cell and Plant isolations.

#### Exiqon biofluids versus Qiagen plasma/serum kits

Triplicate isolations were done for each method from 200 μl plasma. Glycogen was added for all isolations. Manufacturers’ protocols were followed. Triplicate RT-qPCR reactions were performed with material from each isolation to measure endogenous miRs-16 and -150 and exogenous spike-in cel-miR-39. Fold abundance for the Qiagen isolations was calculated against the mean of results for the three Exiqon Biofluids isolations. Separately, for the Qiagen kit, different elution volumes (50 and 100 μl) were compared with the manufacturer-recommended 14 μl.

#### Cerebrospinal fluid RNA isolation

RNA was isolated by the Exiqon Biofluids method with addition of glycogen and spike-in to all isolations. About 50, 100, and 200 μl CSF were used as input volume. Duplicate isolations were performed for each input volume. Higher-abundance miRs-16 and -223 and lower-abundance miR-21 and let-7c were measured in triplicate reactions.

### Reverse transcription and real-time quantitative PCR

Reverse transcription and RT-qPCR steps were performed largely according to the manufacturer’s protocols, with any modifications as described previously.(Witwer et al., 2011, 2012) The stem-loop RT primer design allows specific amplification of mature miRNA; no significant differences are seen in DNase-treated versus untreated samples. Following reverse transcription, samples were diluted with 20 or 30 μl RNase-free water and were mixed, spun down, and stored at −20°C until use. The Applied Biosystems/Life Technologies recommended protocol for qPCR reaction setup was modified for a smaller volume of 10 μl per well before addition of sample, using the same reagent proportions as recommended by the manufacturer. To each well, 2 μl of diluted cDNA was added. Each plate was mixed gently, centrifuged, and loaded into the real-time machine. The manufacturer’s amplification protocol was followed for 45 amplification cycles. Negative controls: no-template and/or no-reverse transcriptase control reactions were performed for selected isolations, substituting water for RNA or the reverse transcriptase enzyme. These reactions consistently failed to amplify or amplified only after 40 PCR cycles on average (data not shown). Reverse transcription and RT-qPCR reagents were from Life Technologies for all experiments except for the Exiqon Biofluids/Qiagen miRNeasy comparison, for which BioRad reagents were used (we conducted a side-by-side comparison of the reagents and found no significant differences in results). Real-time detection systems were the iQ5 and CFX96 instruments (both from BioRad, Hercules, CA, USA).

### Data collection and processing

To obtain Cq values, appropriate thresholds were drawn manually (iQ5) or automatically (CFX96) with checking and manual adjustment by the operator if needed. Mean Cq and standard deviation were calculated for each technical triplicate reaction for each miRNA. Mean Cq and standard deviation (for three isolations) or range (for two isolations) were determined for each type of isolation. Delta Cq values were calculated with reference to the chosen “control” condition for each isolation experiment, as specified above. From these values, fold abundance with respect to the control was calculated (Livak and Schmittgen, 2001). Mean fold abundance values and standard deviations were determined for the examined miRNAs.

## Results

Five RNA isolation methods/kits were compared for isolation of small RNA from human plasma. All but one method was tested in the presence and absence of glycogen as RNA carrier. Because assessment of different RNA isolation methods requires the use of a standard source sample, we conducted all isolations from plasma obtained from a single blood draw from a single donor. This plasma had been centrifuged twice to obtain platelet-poor plasma as described in methods. Aliquots of plasma had been frozen at −80°C and had not been thawed previously.

The low abundance of exRNA necessitates consideration of a carrier molecule. We chose glycogen as a carrier rather than complex exogenous RNA mixtures such as yeast tRNA or MS2 phage RNA. Little has been published on this topic, but there seem to be conflicting opinions on whether biological carrier RNAs might be responsible for non-specific hybridization or amplification in downstream analytics. Highly pure glycogen is an alternative carrier/co-precipitant that may avoid these unresolved questions since it is inert and does not cause reported interference with downstream assays (Turchinovich et al., 2011; Turchinovich and Burwinkel, 2012). Still, since glycogen is purified from biological sources, chiefly bivalves, some commercial glycogen preparations may contain nucleic acids. As a result, UV-treated glycogen or an alternative co-precipitant, linear polyacrylamide (LPA), have been proposed (Bartram et al., 2009). In tests of the glycogen we used, however, we were unable to obtain specific signal for any of the mammalian miRNAs or other small RNAs examined (data not shown).

In addition to glycogen, we used a synthetic spiked-in cel-miR-39 RNA. It is important to remember that the spike-in is useful for assessment of recovery but cannot be used for biological normalization. In the experiments described here, no normalization was necessary, since the same plasma sample was used throughout. As noted in Section “Materials and Methods,” fresh dilutions of cel-miR-39 stock were made for each of three successive isolation experiments; thus, normalization by spike-in between experiments would be inappropriate and was not done.

We first compared the Ambion mirVana kit [based on (Chomczynski and Sacchi, 1987)] with the Exiqon miRCURY Cell and Plant Kit and TRIzol LS extraction followed by mirVana column clean-up. Each of these three protocols was performed in triplicate with and without the use of glycogen as a carrier. The mirVana and TRIzol LS methods include a phase separation step, whereas the miRCURY method does not. Thus, the miRCURY protocol is faster, less technically demanding, and is likely subject to less technical variation, operator-dependent, and otherwise.

To assess performance of these kits, two endogenous miRNAs and the spike-in were measured in triplicate for each isolation replicate by RT-qPCR. (Please observe that we use the term “recovery” or “apparent recovery” to describe results, although from the RT-qPCR results it is not necessarily clear whether differences arise from different RNA recovery efficiencies, different inhibitor removal efficiencies, or both).

**Figure 1:**
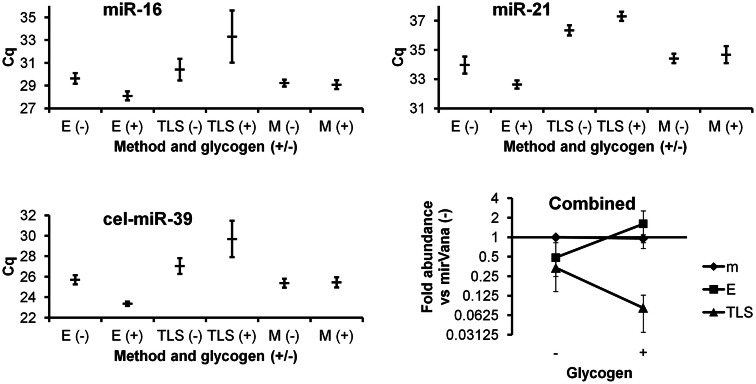
**Comparison of three RNA isolation methods, with and without glycogen**. miRNAs from plasma RNA isolated in triplicate by the three indicated methods (E, Exiqon Cell and Plant kit; TLS, TRIzol LS; M, mirVana isolation kit), with and without glycogen, were assessed by RT-qPCR in triplicate. Results based on two endogenous and one spiked-in exogenous miRNA are shown. Raw Cq averages and standard deviations are presented for the higher-abundance miR-16, lower-abundance miR-21, and spiked-in cel-miR-39. The “combined” panel shows mean apparent combined abundance of miRNAs in samples isolated by the mirVana no-glycogen method set equal to one. Fold abundance (from deltaCq method) is displayed with standard deviation bars.

The Ambion mirVana kit yielded similar results with and without glycogen (Figure 1). Without glycogen, the Exiqon protocol showed slightly lower recovery than both mirVana kit protocols. Addition of glycogen to the Exiqon protocol significantly enhanced recovery by more than threefold compared with the no-glycogen Exiqon method (*p* < 0.02) or by 1.6-fold versus mirVana (trend, with *p* < 0.1). TRIzol isolation followed by column clean-up did not perform as well as the other methods in our hands (Figure 1). Surprisingly, the addition of glycogen appeared to exacerbate the low RNA recovery achieved by this method.

**Figure 2:**
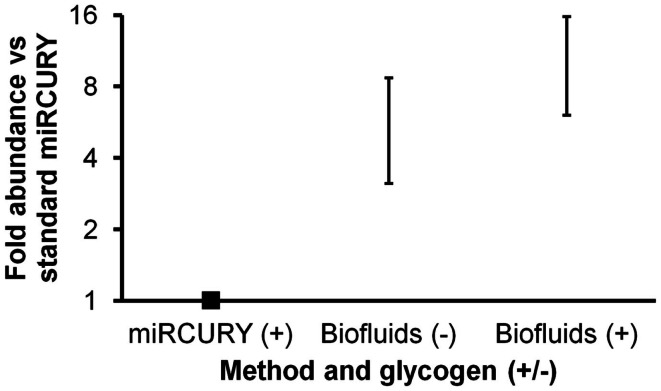
**Comparison of Exiqon miRCURY Cell and Plant and miRCURY Biofluids kits**. Results of plasma miRNA RT-qPCR (technical triplicates) of duplicate isolations by the two Exiqon methods, with miRCURY Cell and Plant conducted with glycogen and Biofluids conducted both with and without glycogen. Fold abundance is relative to Exiqon miRCURY Cell and Plant. Bars represent range. Results of RT-qPCR for three endogenous and one spiked-in miRNA are shown. As noted in the text, the depicted differences versus Exiqon miRCURY Cell and Plant were significant.

A new, biofluids-specific Exiqon kit became available in December, 2012. According to the manufacturer, this kit allowed better RNA recovery as well as PCR inhibitor removal in comparison with other methods. (It should be mentioned that RNA species less than 1000 nt in length are preferentially recovered.) The two protocols are similar in terms of time and operator skill requirements. We performed a second isolation experiment to compare the performance of this Biofluids kit (with and without glycogen) with the standard miRCURY kit (with glycogen). For this experiment, duplicate isolations were performed, followed by triplicate miRNA RT-qPCR reactions for each isolation replicate. Both without and with glycogen, the Biofluids kit displayed significantly greater recovery than standard miRCURY with glycogen (Figure 2, *p* < 0.02 and *p* < 0.01, respectively), with large fold abundance differences averaging about six (without glycogen) or >10 (with glycogen). For qPCR Cq results by all methods, standard deviation of the technical triplicates was typically below 0.1 for high-abundance miRNAs. For the low-abundance miRNA miR-34a, high variability was observed.

In a third experiment, conducted with addition of glycogen for all isolations, the miRCURY Biofluids kit was compared with another biofluids-specific protocol: the Qiagen miRNeasy Serum/Plasma system. The Qiagen protocol includes a phase separation and is thus comparable to mirVana and TRIzol protocols in time and technique requirements. In contrast with the other methods we tested, the Qiagen protocol calls for a very small elution volume (14 μl), with the apparent intent of providing a more concentrated RNA sample.

**Figure 3:**
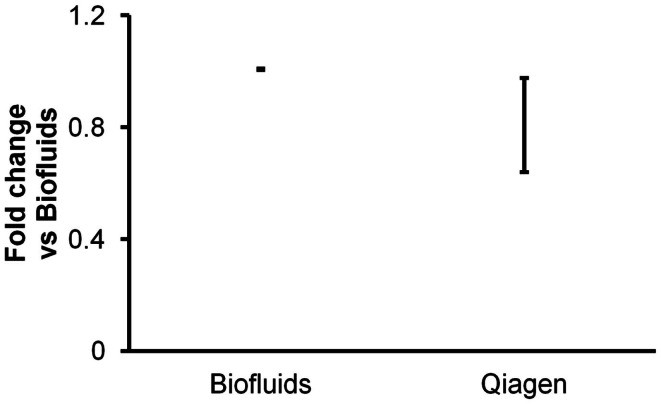
**Comparison of two biofluids-specific kits**. Triplicate plasma RNA isolations with Exiqon Biofluids and Qiagen miRNeasy Serum/Plasma (glycogen added for both) were compared in triplicate RT-qPCR reactions for two endogenous and one spiked-in miRNA. Fold abundance is relative to Exiqon Biofluids. Note that for the Exiqon and Qiagen kits, the eluate volumes were 50 and 14 μl, respectively, as specified by the manufacturers’ protocols, so the results are not necessarily directly comparable (see text).

Triplicate isolations were performed followed by triplicate RT-qPCR measurements of endogenous miRNAs and spiked-in cel-miR-39. As with all other experiments performed for this report, we measured miRNAs in 2 μl of purified RNA eluted as per the manufacturers’ protocols. Both kits produced highly consistent results between isolation replicates. Results indicated similar apparent recovery by the two kits (Figure 3), albeit with slightly lower recover for the Qiagen kit. However, it must be considered that the results were obtained with 2 μl of elution volumes of 50 μl (Exiqon) versus 14 μl (Qiagen). Assuming that RNA was appropriately recovered with a 14-μl elution volume, i.e., that RNA was not left on the column, this might imply lower recovery by the Qiagen kit. Since we and others have previously observed with other isolation kits that smaller elution volume does not necessarily produce more concentrated RNA (Witwer et al., 2013), we performed an additional experiment to determine whether larger elution volume would recover additional RNA from the Qiagen columns. In this experiment, duplicate isolations were performed for 14, 50, and 100 μl elution volumes. Most of the RNA was in fact recovered by the 14-μl volume: practically all of a low-abundance RNA and ∼70–90% of high- to medium-abundance RNAs (data not shown). Thus, the apparent difference in recovery of the Exiqon Biofluids and Qiagen Plasma/Serum kits does not seem to be addressed by altering the elution volume for the latter.

**Figure 4:**
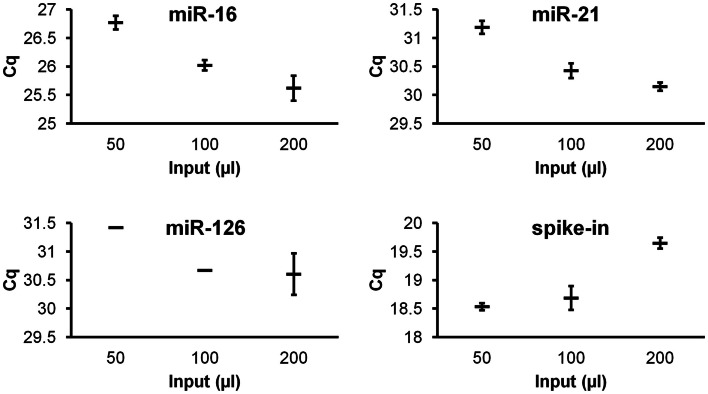
**Quantitative recovery of RNA from plasma does not extend to highest input volume**. The Exiqon Biofluids kit was used to isolate RNA in duplicate isolations for each of the three indicated quantities of plasma. Range of Cq values for higher-abundance miR-16, lower-abundance miRs-21 and -126, and spiked-in cel-miR-39 (same input amount for each isolation) are shown.

**Figure 5:**
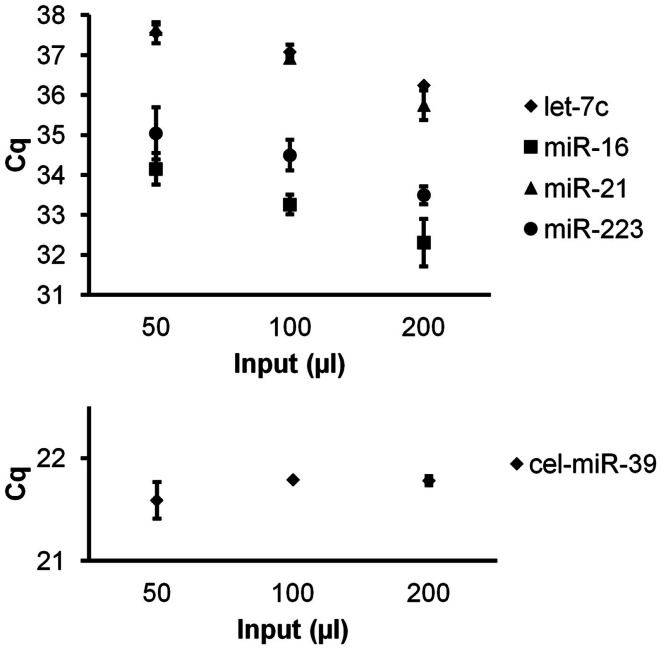
**Quantitative recovery of RNA from cerebrospinal fluid by Exiqon Biofluids**. The Exiqon Biofluids kit was used to isolate RNA in duplicate isolations for each of three quantities of CSF. Linear response to input volume is shown for four endogenous miRNAs as indicated (measured in triplicate for each isolation), as well as spiked-in cel-miR-39 (same input amount for each isolation). Bars represent range of Cq values for the duplicate isolations.

To investigate quantitative recovery of RNA using the Exiqon Biofluids kit, we isolated RNA from 50, 100, and 200 μl of plasma and from the same volumes of *Macaca nemestrina* CSF from a single donor. The same amount of spike-in RNA was added to each isolation. In the case of plasma, endogenous miRNA had greater apparent recovery with increasing input volume, but the increase was not proportional from 100 to 200 μl input (Figure 4). Although we examined only three endogenous miRNAs, the lower-abundance miR-126 in particular displayed no apparent increase in recovery at 200 μl (Figure 4). Interestingly, cel-miR-39 was measured at a lower level in the 200-μl input samples, suggesting inefficient apparent recovery because of column clogging and/or concentration of inhibitors. In contrast, largely quantitative recovery was observed from increasing volumes of input CSF (Figure 5), a fluid that in health is relatively protein-poor compared with plasma. Spiked-in cel-miR-39 was recovered at the same level with each volume of input.

## Discussion

Our results indicate that the Exiqon miRCURY Biofluids Kit outperforms other RNA isolation methods we tested, at least for isolation of small RNAs from plasma. This finding, furthermore, should be viewed in light of the relative ease of execution of the Exiqon method when compared with traditional phase-separation-based methods. Ease of use and short processing time are certainly appropriate considerations when results are not compromised.

Without glycogen, the standard Exiqon miRCURY slightly underperformed, and with glycogen, it slightly outperformed the Ambion mirVana kit. Both kits outperformed TRIzol LS with column clean-up in our hands. The new Exiqon miRCURY Biofluids kit appeared to be superior for small RNA isolation when compared with the standard miRCURY kit. With miRCURY Biofluids, all miRNAs were apparently recovered at higher quantities in the presence of glycogen, although the difference was not statistically significant at an alpha of 0.05. miRCURY Biofluids also provided better recovery than the Qiagen miRNeasy Serum/Plasma kit in our hands, considering the small elution volume from the Qiagen kit.

Although we found that the Qiagen Serum/Plasma kit allowed recovery of most of the on-column RNA with only 14 μl of eluant, it is not clear to us that using smaller elution volumes generally achieves greater RNA concentration. For some protocols and columns, smaller volumes of eluant may simply leave RNA on the column rather than providing greater concentration. An analogy that has been used previously (Eldh et al., 2012, International Society for Extracellular Vesicles Workshop in New York City) likened the column filter to a rag: to rinse it, the rag must be soaked completely (Witwer et al., 2013). Thus, we would recommend that any attempts to concentrate RNA in this manner be tested rigorously. Overall, additional optimization of existing methods is encouraged, since repeated extractions and temperature during isolation (Burgos et al., 2013), different carriers and spike-ins, and elution volumes are among the parameters that could be optimized for specific RNA isolation kits.

A conclusion from our experiments that we found particularly surprising is that TRIzol LS isolation followed by column clean-up did not perform well in our hands. We are uncertain as to why this method performed so poorly in our case or why the addition of glycogen appeared further to decrease yield, since this method or variations on it have been used with excellent results by others. The variability and low yield we report here is likely due to a separate issue from that reported by Kim et al. (2012b), who retracted their *Cell* article in 2012 after observing that standard TRIzol extraction resulted in apparently poor recovery of low-GC-content miRNAs from low-abundance samples. However, clarity on this point has yet to be achieved.

We submit that our results prompt at least three additional recommendations. First, based on our input volume experiments, it would appear that using 200 μl of plasma as input does not necessarily result in quantitatively greater recovery. This echoes previous findings (Kim et al., 2012a). In contrast, RNA was quantitatively recovered from the smallest to the largest input volume of CSF. Thus, it may be important to determine optimal input volume for each biofluid and isolation method. Second, we would like to emphasize the importance of co-isolation of all RNA samples that are that are to be compared within an experiment to avoid introduction of batch effects (as we have noted previously) (Witwer et al., 2011, 2012; Sisk et al., 2012). Although raw Cq values for spike-in and endogenous miRNA measurements were highly consistent within each isolation experiment, they varied somewhat from experiment to experiment. For the spike-in, this may be due at least in part to the fact that we made a new dilution of spike-in for each isolation experiment from a highly concentrated stock. However, it is also clear that batch isolations (and batch RT reactions) should not be compared directly. Third, the Cq differences between replicate isolations, while often half a cycle or less, indicate that technical differences could contribute to apparent differences in miRNA expression, especially in studies with low “n.” Many miRNA publications report differential expression of twofold or less. This underlines the importance of proper numbers of biological replicates and may also indicate that, where possible, multiple isolations from the same sample could increase rigor.

This study has several limitations, which may be helpful to review as they indicate opportunities for advances in the field. We measured a limited number of endogenous miRNAs in this study. It is possible that recovery may differ from one method to another on a miRNA-by-miRNA basis, similar to the differences reported by Kim et al. (2012b), in their retraction letter. This could be due to GC content, length, or other features. Assuming availability of appropriate resources, a miRNome-wide comparison of isolation methods might be highly useful. Also, we have not determined whether the differences we observe are due to inhibitors, to differential RNA recovery, or to some ratio of the two. Additional studies would be needed to make this determination. Many RNA isolation kits are available, along with many method modifications, and we have explored only a small portion of this methods space. We encourage other researchers to join us in comparing methods and working toward standardization as well as improvement of existing practices. Finally, we have examined only whole platelet-poor plasma in our methods comparisons. Other biofluids are also important potential sources of biomarkers, and comparatively little methodologic information is available for most biofluids [see, however (Burgos et al., 2013), published during review of this paper]. It is also possible that different isolation methods are better suited to recovering RNA from specific sub-populations of the various carrier particles that protect RNA in plasma and other biofluids. For example, isolation methods for exosomal RNA have been rigorously compared (Eldh et al., 2012). Similar studies for other RNA carriers would be informative and would help to establish whether different methods are needed, e.g., for isolation from whole plasma versus extracellular vesicles or protein complexes.

## Conflict of Interest Statement

This research was conducted in the absence of any commercial or financial relationships that could be construed as a potential conflict of interest. Qiagen provided a sample of the miRNeasy Serum/Plasma Kit at no cost, as noted in the Acknowledgments, but did not influence the execution or reporting of this study in any way.
